# Alterations to neuromuscular properties of skeletal muscle are temporally dissociated from the oxygen uptake slow component

**DOI:** 10.1038/s41598-020-64395-5

**Published:** 2020-05-07

**Authors:** Trishan Gajanand, Sonia Conde Alonso, Joyce S. Ramos, Jean-Philippe Antonietti, Fabio Borrani

**Affiliations:** 1 Department of Exercise Sciences, Faculty of Science, University of Auckland, Auckland, New Zealand; 2 Institute of Sport Sciences of University of Lausanne (ISSUL), Faculty of Biology and Medicine, University of Lausanne, Lausanne, Switzerland; 3 SHAPE Research Centre, Exercise Science and Clinical Exercise Physiology, College of Nursing and Health Sciences, Flinders University, Bedford Park, South Australia 5042 Australia; 4 Institute of Psychology, University of Lausanne, Lausanne, Switzerland; 5 School of Human Movement and Nutrition Sciences, The University of Queensland, St Lucia, Queensland Australia

**Keywords:** Respiration, Energy metabolism

## Abstract

To assess if the alteration of neuromuscular properties of knee extensors muscles during heavy exercise co-vary with the SCV (${\dot{{\rm{V}}}{\rm{O}}}_{2}$ slow component), eleven healthy male participants completed an incremental ramp test to exhaustion and five constant heavy intensity cycling bouts of 2, 6, 10, 20 and 30 minutes. Neuromuscular testing of the knee extensor muscles were completed before and after exercise. Results showed a significant decline in maximal voluntary contraction (MVC) torque only after 30 minutes of exercise (−17.01% ± 13.09%; p < 0.05) while single twitch (PT), 10 Hz (P10), and 100 Hz (P100) doublet peak torque amplitudes were reduced after 20 and 30 minutes (p < 0.05). Voluntary activation (VA) and M-wave were not affected by exercise, but significant correlation was found between the SCV and PT, MVC, VA, P10, P100, and P10/P100 ratio, respectively (p < 0.015). Therefore, because the development of the SCV occurred mainly between 2–10 minutes, during which neuromuscular properties were relatively stable, and because PT, P10 and P100 were significantly reduced only after 20-30 minutes of exercise while SCV is stable, a temporal relationship between them does not appear to exist. These results suggest that the development of fatigue due to alterations of neuromuscular properties is not an essential requirement to elicit the SCV.

## Introduction

At the onset of constant power exercise, the muscles requirements for ATP re-synthesis increase immediately following exercise onset. The same cannot be said about the oxygen uptake ($\dot{{\rm{V}}}$O_2_) response that instead, displays a sluggishness to fully activate metabolism^1–3^. During exercise below the lactate threshold, $\dot{{\rm{V}}}$O_2_ rises mono-exponentially to a new steady-state^3,4^ and from unload pedalling, the rise of $\dot{{\rm{V}}}$O_2_ increases as a linear function of work-rate^5^. However, during constant-load exercise completed at intensities above the lactate threshold, the $\dot{{\rm{V}}}$O_2_ response becomes more complex with a second rise in $\dot{{\rm{V}}}$O_2_, developing slowly, which is superimposed onto the initial $\dot{{\rm{V}}}$O_2_ response^6^. This slowly developing rise in $\dot{{\rm{V}}}$O_2_, termed the slow component of $\dot{{\rm{V}}}$O_2_ (SCV), results in a greater end-exercise $\dot{{\rm{V}}}$O_2_ than that predicted by the sub-LT $\dot{{\rm{V}}}$O_2_-power output relationship. It has been proposed that the inefficiency which leads to the SCV originates primarily from the active muscles^7^. However, the reason for this observed inefficiency in the muscle is not clear and may potentially result from reduction of ATP production per mole of oxygen (P/O ratio), diminution of the energy yield per unit of hydrolysed ATP, alteration of neuromuscular properties of muscle filament to produce force, and/or deterioration of the motor pattern of the motion^8^. However, the potential link between the alteration of neuromuscular properties of muscle filament and progressive muscle inefficiency, and therefore the SCV, is not well explored. The capability of muscle to produce force progressively declines during high-intensity exercise when fatigue gradually develops^9^. It is widely accepted that alterations of the metabolic milieu of locomotor muscles are mainly responsible for the decline in force. Indeed, neuromuscular properties of knee extensor muscles are sensitive to the accumulation of muscle metabolites such as adenosine diphosphate (ADP), inorganic phosphate (P_i_), hydrogen ion (H^+^), and magnesium ion (Mg^2+^). Muscular force production is reduced by increases in [P_i_], [Mg^2+^], and [H^+^] while augmented by an increase in [ADP]^10^. Additionally, increased [ADP] reduces cross-bridge cycling rate^10^.

Since the SCV occurs during high-intensity exercise, and because high-intensity exercise is always associated with changes in metabolite concentration that may produce an alteration in neuromuscular properties of muscle filament, the latter may be considered a putative mediator of the SCV (in line with current views;^11–14^).

Standardised investigative methods of neuromuscular function, such as peripheral nerve stimulation (PNS), have been extensively used to explore the complex relationship between exercise and fatigue. For instance, using PNS, Decorte and colleagues showed that during exhaustive constant-load cycling at 80% of maximum aerobic power output, neuromuscular properties were significantly reduced as early as 20% of the total duration of cycling, indicating a potential link with the SCV^15^. Although, little is known about the possible relationship between the SCV and the alteration of neuromuscular properties of knee extensors, Keir and colleagues^16^ in 2016 showed a significant association between the decrements in muscle torque and the SCV, without changes in muscle activation over the course of the exercise. Also in an *in vivo* study using cycle ergometry, Cannon and colleagues have shown that changes in velocity-specific peak power generated in the initial minutes of exercise were correlated to the SCV measured between three and eight minutes of heavy and severe exercise^17^. Results from the same working group suggest that the SCV during heavy exercise arises from both contractile and mitochondrial sources^17^. Furthermore, using self-paced dynamic concentric extension/flexion of the knee and interleaving voluntary and electrically evoked contractions, Froyd and colleagues have shown, even without measuring directly VO_2_ kinetics, that fatigue progresses with similar dynamics to those expected of the SCV during an approximately 6-min time trial^18^. However, these findings do not show the mechanism linking the alteration of neuromuscular properties of knee extensors, per se, and the SCV.

The aim of the present study was to quantify the alteration of neuromuscular properties of knee extensors during heavy exercise and to see if these impairments co-vary, as function of time, with the SCV amplitude. The hypothesis was that the SCV amplitude correlates with the change in neuromuscular properties of knee extensor muscles, depicted by a decrease in evoked peak torque.

## Methods

### Participants

Eleven healthy, recreationally active, male participants (mean ± SD, age 27 ± 6.6 years, body mass 76 ± 7.6 kg, and height 179 ± 8.1 cm) were recruited for this study. The participants were provided with a participant information sheet outlining the procedures involved, time commitment, and requirements of the study. Participants were screened using a self-administrated pre-exercise health questionnaire designed to identify those who may be at risk of an adverse event during exercise. Participants were advised of their right to withdraw from the study at any time without disadvantage.

Participants were asked to avoid, in the 24 h preceding a testing session, strenuous physical activity, alcohol, tobacco, and caffeine. Furthermore, participants were asked not to consume any food for the 3 h preceding a test and to arrive fully hydrated. All tests were completed at a similar time of day (±1 h). The study was approved by the local humans Ethics Committee and conformed to the latest revision (2013) of the Declaration of Helsinki. All participants provided written informed consent prior to participation.

### Experimental design

**Figure 1:**
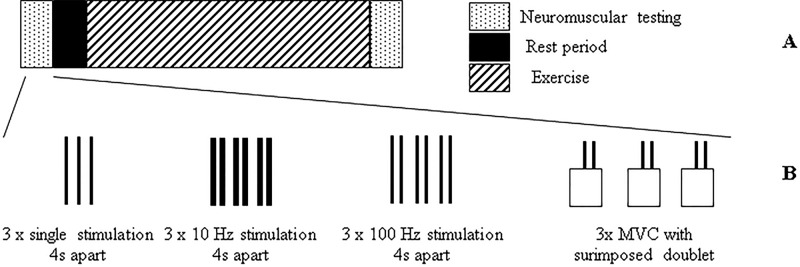
Description of events completed during experimental testing (**A**) and during neuromuscular testing (**B**). Neuromuscular tests (dotted box) were completed prior to the rest period (filled box) and after exercise (box with diagonal lines). Neuromuscular testing involved three single stimulations (single solid lines) followed by three stimulations at 10 Hz (thick-double solid lines) and then three stimulations at 100 Hz (thin-double solid lines). Each stimulation had a four second separation. Finally, three MVCs were completed with superimposed 100 Hz doublets applied (empty box with thin-double solid lines), each separated by a minute rest period.

This study involved each participant attending six separate laboratory sessions, with at least a 48 h interval between tests, over a three-week period. All tests were completed in an air-conditioned (21 °C ± 1 °C) exercise physiology laboratory. The first session involved an incremental ramp test on a cycle ergometer (Velotron, RacerMate, Seattle, WA, USA). This test was used to assign a work-rate for the subsequent five experimental sessions during which constant work-rate exercise was completed. Following the incremental ramp test, participants were familiarised with the procedure to be used to evaluate neuromuscular function. The five experimental sessions (Fig. 1A) involved participants cycling for different durations of time in a random order at an identical power (heavy domain, see below). Neuromuscular evaluation was performed before exercise, and within 1-minute of completing constant work-rate exercise. This was completed to determine the central and peripheral fatigue through neural and neuromuscular properties of the knee extensor muscles.

### Testing procedures

#### Incremental ramp test

Incremental ramp exercise test was completed in order to determine the gas exchange threshold (GET) and peak oxygen consumption ($\dot{{\rm{V}}}$O_2peak_). After a three-minute rest period seated on the cycle ergometer, participants performed six minutes of baseline cycling at 60 watts, after which, the work rate was increased by the rate of 30 watts each minute until reaching the limit of tolerance. The ergometer allows participants to cycle at a constant power output independent of pedal rate, though participants were asked to maintain a pedal rate of 85 revolutions per minute (rpm). Verbal encouragement was provided throughout the test. The test was terminated when the pedal rate dropped by more than 10 rpm (i.e. 75 rpm). All cycle tests were completed on an electromagnetically braked cycle ergometer where the seat and handlebars were fully adjustable both vertically and horizontally. The horizontal and vertical direction of both the seat and handlebars were adjusted to suit each participant and were recorded following the ramp test and replicated for subsequent tests. Pulmonary gas exchange and ventilation were measured from the beginning of the rest period until cessation of the test.

#### Step transition tests

Each participant attended a total of five experimental sessions during which cycling at a constant-load were completed. The test began with a 5-minute rest period before participants completed three minutes of unloaded cycling (20 watts). At the end of the three minutes, an immediate transition to the work rate equal to 30%∆ (GET plus 30% of the difference between the work rate at the GET and $\dot{{\rm{V}}}$O_2peak_; heavy exercise) was imposed with the duration altered at each session (2, 6, 10, 20, 30 minutes). Constant power was maintained at 85 rpm and was maintained for the duration specified for each of the tests.

#### Neuromuscular evaluation

Neuromuscular evaluation (Fig. 1B) consisted of (1) 3 x single supra maximal electrical stimulations, each separated by four seconds, (2) 3 x paired at 10 Hz (two stimulation pulses separated by 100 ms) and 3 x paired at 100 Hz (two stimulation pulses separated by 10 ms) electrical stimulations, each separated by four seconds, and (3) 3 x five-second isometric maximal voluntary contraction (MVC) tests of the knee extensor muscles during which a 100 Hz doublet was superimposed to the MVC. A one-minute rest period separated each MVC. Strong, standardised, verbal encouragement was provided throughout the MVC. In order to increase the contact between the electrode and the skin during all electrical stimulations, a pressure was applied to the cathode electrode using a wooden handle with a rubber end. Note that during post exercise, each sequence was repeated only one time in order to diminish the possible effect of recovery time. Less than one minute was required to position the participant for testing after exercise.

### Measurements

#### Pulmonary gas exchange

During all tests, pulmonary gas exchange was continuously measured using a computerised system (MetaMax 3b, Cortex, Leipzig, Germany). The system used an infrared sensor and an electrochemical cell to measure fractional concentrations of CO_2_ and O_2_ in expired gas. A digital transducer turbine assessed inspired and expired gas volume. A capillary line was used to continuously sample gas concentration. The transducer and the capillary line were securely attached to the facemask, which was firmly fitted to the participants face using Velcro straps. Immediately before each exercise test, the gas analysers were calibrated with gases of known concentration (O_2_ = 14.01%, CO_2_ = 6.03%), and the turbine volume transducer was calibrated using a three-litre Rudolph syringe (Cortex, Leipzig, Germany).

$\dot{{\rm{V}}}$O_2peak_ was noted as the highest 30-second average value attained during the incremental ramp test. The GET was determined using a number of measurements: (1) visual examination for the first disproportionate increase in CO_2_ production ($\dot{{\rm{V}}}$CO_2_) from $\dot{{\rm{V}}}$CO_2_ versus $\dot{{\rm{V}}}$O_2_ graph, (2) an increase in ventilatory equivalent of oxygen ($\dot{{\rm{V}}}$_E_/$\dot{{\rm{V}}}$O_2_) without increase in ventilatory equivalent of carbon dioxide ($\dot{{\rm{V}}}$_E_/$\dot{{\rm{V}}}$CO_2_), and (3) increase in partial pressure of end-tidal oxygen with no decrease in partial pressure of end-tidal carbon dioxide. Subsequently, the work rate that would require 30%∆ was calculated and assigned for the experimental tests after accounting for the mean response time for $\dot{{\rm{V}}}$O_2_ during ramp exercise (2/3 of the ramp rate was subtracted from the work rate at the gas exchange threshold and $\dot{{\rm{V}}}$O_2peak_, i.e. 20 watts)^19,20^.

#### PNS

Electrical stimulation was delivered using a high-voltage stimulator (model DS7, Digitimer Stimulator, Hertfordshire, UK). Low intensity stimulation (~ 20 mA) was used to locate the femoral nerve by means of a cathode ball electrode (0.5 cm diameter) which was manually pressed into the femoral triangle and maneuvered until the femoral nerve was properly located (determined by observing contraction of the leg). A 5 cm diameter cathode electrode (American Imex, CA, USA) was then placed on the site after it was cleaned with an alcohol wipe. The anode, a rectangular electrode (18 × 7 cm, American Imex, CA, USA), was placed opposite the cathode in the gluteal fold. Both the cathode and anode electrodes were worn during exercise and therefore both were taped to the skin using micropore tape (3 M Micropore, St. Paul, MN, USA) to limit movement. To determine maximal stimulation, single electrical stimulations (rectangular pulse, 1 ms duration, 400 V) were delivered to the nerve and progressively increased until a plateau in the twitch torque and M-wave amplitude were achieved. The current that achieved plateau was increased by 20%, which was then used for subsequent tests.

#### Torque measurement

The evaluation of neuromuscular function was conducted on the right knee extensor muscles with participants seated in a Biodex isokinetic dynamometer (Biodex Medical Systems Inc., Shirley, NY, USA). The hip and knee angles were fixed at 90° (0° = full knee extension) with the ankle strapped to the lever arm of the Biodex. The rotational axis of the dynamometer was aligned with the lateral epicondyle of the femur, found after palpation. Two crossover straps were placed firmly across the shoulders to limit upper body movement and one strap was placed midway across the thigh of the right leg. Participants were asked to cross their arms across their chest during testing. Adjustments made to the seat position and to the lever arm of the Biodex were recorded for each participant during familiarisation and reproduced for subsequent tests.

#### Electromyography recordings

Once participants were seated, the right vastus medialis (VM) and vastus lateralis (VL) muscles were palpated and prepared for electromyogram signal (EMG) recording. To reduce impedance, the skin around the belly of the muscles was shaven, lightly abraded (3 M Red Dot Trace Prep, Ontario, Canada) and cleaned using 70% isopropyl alcohol wipes (Kendall Company, Mansfield, MA, USA). One pair of silver-chloride electrodes (3 M Red Dot, St. Paul, MN, USA) of 10 mm diameter with an interelectrode (center to center) distance of 2 cm were then placed lengthwise over the prepared muscle. The ground electrode was placed over the patella of the right leg. EMG and torque signals were recorded through chart software (v. 5.5.6, ADInstruments, Sydney, Australia). EMG signals were amplified with a bandwidth frequency ranging from 1.5 Hz to 2 kHz (common mode rejection = 90 dB; impedance = 100 MΩ; gain = 1000). The myoelectric and mechanical responses were digitised on-line at a sampling frequency of 2000 Hz and stored for off-line analysis.

### Data analysis

#### Oxygen uptake kinetic analysis

The breath-by-breath $\dot{{\rm{V}}}$O_2_ data from each of the 30%∆ tests were initially examined to exclude errant breaths caused by coughing, swallowing, sighing, etc., and those values lying more than three standard deviations from the model $\dot{{\rm{V}}}$O_2_ were considered outliers and were removed. The breath-by-breath data from the different exercise durations were subsequently linearly interpolated to provide second-by-second values, and, for each individual, repetitions from different durations were time aligned to the start of exercise and the ensemble averaged. The primary component (phase 2) kinetics were isolated to identify the mono-exponential region and modelled by the following equation:

$$\dot{V}{O}_{2}(t)=\dot{V}{O}_{2b}+{A}_{p}\cdot (1-ex{p}^{\left(-\frac{t-t{d}_{p}}{{\tau }_{p}}\right)})\cdot U$$

Where $\dot{{\rm{V}}}$O_2_(t) represents $\dot{{\rm{V}}}$O_2_ at a given time t; U= 0 for t < td_1_ and U = 1 for t ≥ td_1_

$\dot{{\rm{V}}}$O_2b_ is the $\dot{{\rm{V}}}$O_2_ during unloaded cycling defined as the mean $\dot{{\rm{V}}}$O_2_ measured over the final 90 seconds of baseline pedaling; A_P_ is the asymptotic amplitudes for the primary phases; τ_P_ is the time constant, and td_P_ represents the time delay. Since the focus of this study was the SC, the cardiodynamic phase was removed from analysis^21,22^, and therefore, not modelled, in order to ensure that the early initial component did not influence the results^23^. Initially, the fitting window extended from 20 seconds (i.e., at the end of phase I) to 80 seconds (only 60 s into the exercise). The window was lengthened iteratively in order to attain four series of the initial window length. For each window length, the parameters of the model were determined with an iterative procedure by minimising the sum of the mean squares of the differences between the model $\dot{{\rm{V}}}$O_2_ and actual $\dot{{\rm{V}}}$O_2_.

Identification of the end of the primary phase was completed using H.B. Rossiter criteria consideration^24,25^.

As such, the amplitude of the slow component at time 2, 6, 10, 20, and 30 minutes were assigned the value (*A*_SX_) and were defined as the difference between the value of $\dot{{\rm{V}}}$O_2_ at a given time and the sum of the primary phase and the $\dot{{\rm{V}}}$O_2b_ at the same given time.

SCV was also described as a percentage of the primary component (SCV%) since this ratio would provide information regarding the loss of efficiency.

#### Neuromuscular function analysis

From the EMG trace of single stimulations, peak-to-peak amplitude (M-waves) of the VL (MWVL) and VM (MWVM) were measured. Peak torque (PT) was determined from the torque signal of the single twitch. The highest torque achieved during MVC in their respective conditions were taken as the MVC torque. The PT of doublet stimulations were quantified and termed P10 and P100 for 10 Hz, and 100 Hz, respectively. In addition, the P10-to-P100 ratio (P10/P100) was calculated to assess for the occurrence of low or high frequency fatigue.

The voluntary activation (VA) level was calculated by expressing any increment in torque evoked during maximal isometric contractions (superimposed twitch) as a fraction of the amplitude of the response evoked by the potentiated doublet^26^.

In agreement with the work by Strojnick and colleagues, the following correction factor (CF, the ratio between the torque just before the superimposed doublet divided by MVC peak torque) was used in order to take into account the possibility that the superimposed twitch was not necessarily applied when the torque level was at the true maximal voluntary force^27^.

$$VA=100-\left[CF\cdot \left(\frac{superimposed\,doublet}{potentiated\,doublet}\right)\right]\cdot 100$$

All data presented are the average of three measurements in pre, and a single measurement on post.

### Data and statistical analysis

Data were normalised by expressing the measures taken immediately after exercise as a percentage change relative to before exercise. This was completed to avoid day-to-day variations in measures that may occur. Normality test (Kolmogorov-Smirnov) and F-test of equality of variances were completed to test for normal distribution and equality of variance. One-way repeated measures analysis of variance (ANOVA) was used to test the effect of exercise duration on measurers of neuromuscular function. When a significant main effect was found, significant differences were located using Tukey’s post hoc analysis test. Pearson correlation coefficient was used to assess relationships between the change of SCV% and changes to neuromuscular parameters. Analyses were completed with Box and Tidwell tests, and the Theil method (Theil nonparametric regression technique). The Box and Tidwell test assesses whether the association between the slow component and fatigue is linear or not, and therefore related to time. In contrast, Theil’s regression highlights, in a qualitative way, the points that are distant from the linear relationship. For all tests, significance was set at p < 0.05. Data are expressed as mean ± SD.

### Ethical approval

The University of Auckland Human Participants Ethics Committee approved this study. Written informed consent was provided by all participants prior to participation. All procedures conformed to the latest revision (2013) of the Declaration of Helsinki.

## Results

### Oxygen uptake kinetics

Mean $\dot{{\rm{V}}}$O_2peak_ was 3.95 ± 0.18 l^.^min^−1^ and the mean power output corresponding to 30%∆ was 200 ± 11 watts. During the three minutes of unloaded pedalling at 85 rpm, $\dot{{\rm{V}}}$O_2b_ reached a value of 0.85 ± 0.19 l^.^min^−1^. Asymptotic amplitudes of the primary phase attained 1.85 ± 0.38 l^.^min^−1^ with a time constant of 27.1 ± 15.0 s and a time delay of 12.8 ± 2.3 s. Amplitude of the slow component at time 2, 6, 10, 20, and 30 minutes are presented in Table 1. Values of SCV as a percentage of the primary component are also described.

**Table 1:** Time course of slow component amplitude in absolute, and in percentage of the primary component.

	Amplitude (l^.^min^−1^)	Amplitude (% of Ap)
AS_2_	0.037 ± 0.056	1.9 ± 2.5
AS_6_	0.298 ± 0.130*	16.6 ± 8.6*
AS_10_	0.373 ± 0.150*	20.9 ± 10.2*
AS_20_	0.450 ± 0.202*&	25.3 ± 13.2*&
AS_30_	0.452 ± 0.246*&	29.1 ± 16.3*&%

AS2, AS6, AS10, AS20, and AS30 are the amplitude of the slow component at time 2, 6, 10, 20, 30 min respectively. Ap is the amplitude of primary component. *Significantly different from 2 min. &: Significantly different from 6 min. %: Significantly different from 10 min. Data are presented as mean ± SD.

### Neuromuscular function

MVC measurement showed alteration over the course of exercise (Table 2). Post-hoc test revealed a significant reduction after 30 minutes of cycling compared to before exercise, 2, 6, and 10 minutes of exercise. After 20 minutes of exercise, a trend towards significance was observed compared to before exercise (*p* = 0.1) No effect of exercise duration was detected for VA (Table 2; *p* > 0.05).

**Table 2:** Changes in neuromuscular function over the time course of the slow component.

	2 min	6 min	10 min	20 min	30 min
MVC [%]	−2.81 ± 5.39	−2.32 ± 4.19	−2.87 ± 5.31	−9.26 ± 9.67	−17.01 ± 13.09^*^
VA [%]	−1.81 ± 3.14	0.18 ± 3.90	−1.59 ± 3.4	−2.72 ± 3.92	−4.32 ± 5.66
MWVM [%]	−1.17 ± 5.72	−2.86 ± 8.28	−0.52 ± 8.50	−4.21 ± 8.05	−8.23 ± 7.59
MWVL [%]	4.07 ± 10.33	6.91 ± 6.39	5.07 ± 8.43	8.32 ± 15.07	6.9 ± 28.92

MVC: maximal voluntary contraction, VA: voluntary activation, MWVM: M-wave amplitude of vastus medialis, and MWVL: M-wave amplitude of vastus lateralis. *Different from base line, 2 min, 6 min, and 10 min (p < 0.05). Data are presented as mean ± SD.

No effect of exercise duration was detected for the M-wave amplitude of the VM and VL muscles (Table 2).

**Figure 2:**
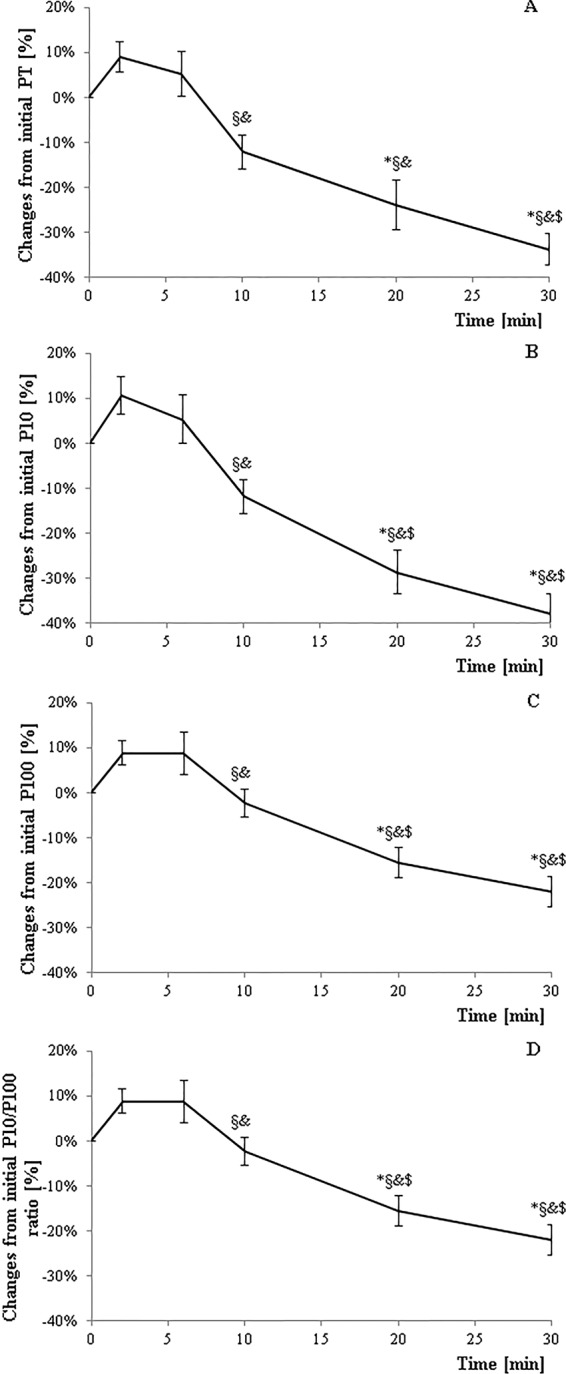
Neuromuscular alterations for peak twitch amplitude (**A**), 10 Hz paired (P10) stimulation (**B**), 100 Hz paired (P100) stimulation (**C**), and P10/P100 (**D**) over the course of exercise. *Significant difference from baseline (p < 0.05); ^§^Significant difference from 2 minutes. (p < 0.05). ^&^Significant difference from 6 minutes (p < 0.05). ^$^Significant difference from 10 minutes (p < 0.05). Error bars are SE.

Twitch amplitude (Fig. 2A) showed a significant reduction at 30 minutes of exercise compared to before, 2, 6, and 10 minutes of exercise; (*p* < 0.05). A significant reduction was also observed after 20 minutes of exercise compared to before, 2, and 6 minutes of exercise (P < 0.05). Finally, significant differences were observed for 10 minutes of exercise compared to 2 and 6 minutes of exercise (*p* < 0.05).

P10 (Fig. 2B) and P100 (Fig. 2C) evolved in a similar manner. Specifically, significant differences were observed for 20 and 30 minutes compared to before, 2, 6, and 10 minutes of exercise (p < 0.05). Furthermore, significant differences were observed at 10 minutes compared to 2 and 6 minutes (*p* < 0.05).

Significant differences for the P10/P100 ratio (Fig. 2D) were found for most exercise durations. A significantly lower P10/P100 ratio was observed at 30 minutes compared to before, 2, 6, and 10 minutes of exercise (P < 0.05). After 20 minutes of exercise, differences were observed compared to before, 2, and 6 minutes of exercise (p < 0.05). Furthermore, significant differences were observed at 10 minutes compared to before and 2 minutes of exercise (*p* < 0.05).

### The SCV and fatigue

**Figure 3:**
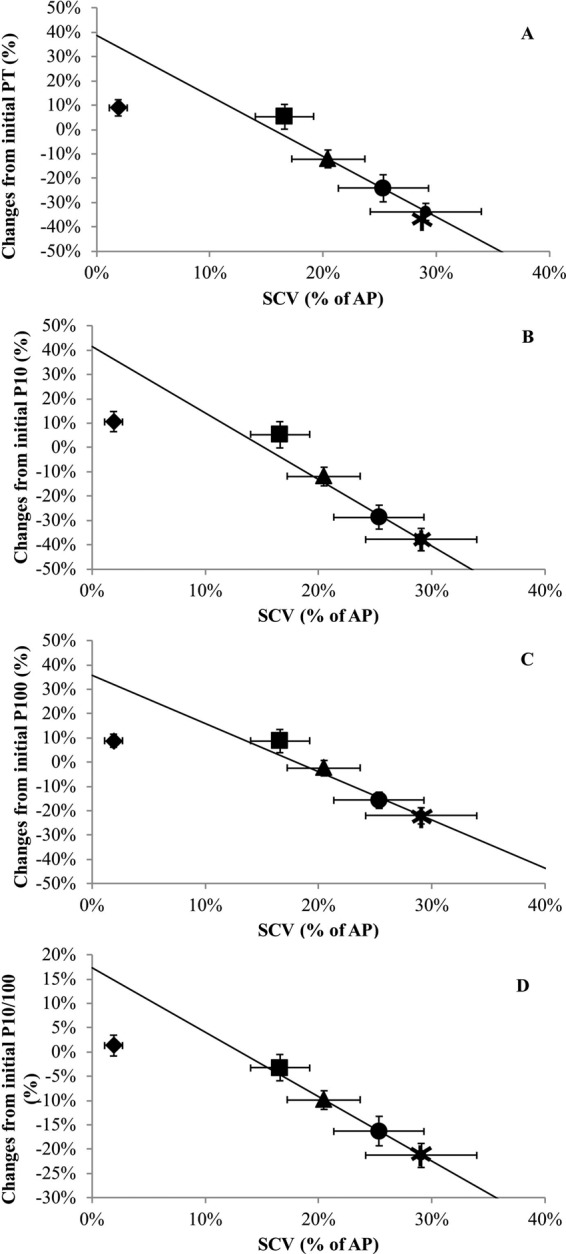
The relationship between peak twitch amplitude (**A**), 10 Hz paired (P10) stimulation (**B**), 100 Hz paired (P100) stimulation (**C**), and P10/P100 (**D**) and the change in SCV relative to the primary phase.◆ 2 minutes; ■ 6 minutes; ▲ 10 minutes; ● 20 minutes; and ✶ 30 minutes represent average values. Theil’s line is characterised by the dashed line. Error bars are SE. Error bars in the figures are presented as SE for more clarity.

Correlation analysis was used to investigate relationships between the SCV% and neuromuscular parameters (Table 3). Changes in M-wave amplitude for either of VL and VM, VA and MVC did not correlate with changes of the SCV relative to the primary phase. However, significant correlations were found between the SCV% and PT, P10, P100 (tendency), and P10/P100 ratio. For these neuromuscular parameters, P10/P100 showed the strongest correlation with SCV% (R^2^ = 0.88), followed by P10 (R^2^ = 0.81), PT (R^2^ = 0.81), and P100 ratio (R^2^ = 0.72). In contrast, the Box and Tidwell’s test was smaller than 0.05 (see Table 3) for correlation relationships suggesting that the relationship is non-linear and therefore unrelated over time. In addition, Theil’s line (see Fig. 3) showed that during the first phase, only the slow component grew (the points of this phase are distant from Theil’s line); while during the second phase, the slow component continued to grow but fatigue also grew (the points of this phase then line up with Theil’s line).

**Table 3:** Correlation coefficient and Box-Tidwell test between the slow component amplitude, as a percentage of the primary phase, and neuromuscular function.

	Correlation		Box-Tidwell test	
	R	P	Z	P
MWVM	−0.69	0.196	−1.70	0.089
MWVL	0.76	0.137	−0.20	0.841
PT	−0.90	0.038	−3.06	0.002
MVC	−0.72	0.172	−5.57	<0.001
VA	−0.52	0.370	−5.48	<0.001
P10	−0.90	0.036	−3.39	<0.001
P100	−0.85	0.065	−3.97	<0.001
P10/P100	−0.94	0.019	−4.50	<0.001

MWVM: M-wave amplitude of vastus medialis, MWVL: M-wave amplitude of vastus lateralis, PT: Peak Torque of the single twitch, MVC: maximal voluntary contraction, VA: voluntary activation, P10: peak torque at 10 Hz doublet stimulation, P100: peak torque at 100 Hz doublet stimulation, P10/P100: ratio of peak torque between 10hz and 100hz doublet stimulation, R: correlation coefficient, Z score statistic, P: significance.

## Discussion

The main finding from the present study was that alterations to force production by the knee extensor muscles were present during exercise at an intensity of 30%∆, which correlated with the development of the SCV, however, a temporal relationship between the development of the SCV and fatigue does not appear to exist.

### Origin of fatigue observed during exercise

With neuromuscular fatigue defined as a reduction in force generating capacity^28^, loss of MVC torque is used as a general index for evaluating the extent of neuromuscular fatigue. In the present study, MVC torque, compared to the beginning of exercise, was found to be significantly reduced only after 30 minutes of cycling at 30%∆. However, while loss of MVC torque is a general index of fatigue, it does not provide information regarding the site of alterations (i.e. neuromuscular fatigue etiology). To determine the origin of the neuromuscular fatigue caused by various durations of cycling at 30%∆, electrical stimulations were delivered at rest, as well as during MVC, allowing for the evaluation of VA, action potential transmission and propagation, and neuromuscular properties. VA, which is commonly used to evaluate central fatigue^29^, was not significantly affected by any exercise durations in the present study. The absence of significant central fatigue suggests that declines in motivation, afferent feedback, or central drive were not present, or that declines in central drive was countered by increased motivation^10,30^. It subsequently suggests a peripheral origin for the induced neuromuscular fatigue. Muscle membrane excitability and neuromuscular propagation appeared to be well preserved, as highlighted by the lack of alterations in VL and VM M-wave amplitudes. In contrast, reductions in evoked forces suggests the presence of peripheral fatigue. Interestingly, signs of peripheral fatigue were observed following shorter exercise durations, suggesting that evoked forces might be more sensitive than MVC for detecting fatigue when it is of peripheral origin. Indeed, PT, P10 and P100 were already reduced after 20 minutes of cycling compared to the beginning of exercise. As M-wave amplitudes were unaltered at all-time points, reductions in evoked forces can highlight either alterations in sarcoplasmic reticulum Ca^2+^ handling^8^ or alterations occurring at the cross-bridge level such as reduced myofibrillar Ca^2+^ sensitivity, and/or reduced capacity for cross-bridge to produce force^31,32^. Further supporting excitation-contraction failure, the P10/P100 ratio was found to be reduced following 10, 20, and 30 minutes of exercise compared to the start of exercise suggesting the presence of low frequency fatigue^33^. A study completed on rat *gastrocnemius* muscle ascribed low-frequency fatigue to Ca^2+^ handling alterations rather than to processes occurring at the cross-bridge level^34^. Indeed, altered Ca^2+^ handling is believed to occur with P_i_ accumulation during the development of fatigue and its subsequent precipitation with Ca^2+^ within the sarcoplasmic reticulum^35^. However, the exact mechanisms responsible for low-frequency fatigue remain unclear as previous results, also obtained using rodents, showed that the site (i.e. Ca^2+^ handling *vs*. cross-bridge level) responsible for this low-frequency fatigue is dependent on the antioxidant status of the individual^36^. Therefore, based on the measures in the present study, it is likely that the observed neuromuscular fatigue following 20 and 30 minutes of cycling at 30%∆ is a result of peripheral rather than central fatigue. Based on the literature, while speculative, it suggests that fatigue it is from either impaired Ca handling or reduced cross-bridge kinetics.^31,32^.

### The SCV and fatigue

Significant correlations were found between the SCV% and PT, P10, P100, and P10/P100. This finding is supportive of the theory regarding the presence of fatigue required to elicit the SCV. In contrast, for these parameters, the Box and Tidwell’s test showed that the relationship between the development of the SCV and the alterations of the neuromuscular properties of knee extensor muscles were non-linear and therefore unrelated over time. In addition, Theil’s line (see Fig. 3) showed two distinct phases; the first phase where only the slow component grew (the points of this phase are away from Theil’s line); while during the second phase, the slow component continued to grow but fatigue also grew (the points of this phase then line up with Theil’s line). (see Fig. 3). In other words, the development of the SCV, in fact, occurred mainly between 2–10 minutes during which neuromuscular properties were relatively stable (only a reduction in the P10/100 ratio was observed after 10 minutes of cycling). In contrast, PT, P10 and P100 were significantly reduced only after 20-30 minutes of exercise compared to baseline values. These results suggest that the development of fatigue due to alterations of neuromuscular properties is not an essential requirement to elicit the SCV at least during the first 10 minutes of exercises. This finding is in line with those from Thistlethwaite and colleagues^37^. They showed that, during heavy cycling exercise, when preceded either by heavy exercise or by heavy knee extensions (requiring twofold greater muscle activation relative to heavy exercise), τ_p_, gain of the primary response, and the amplitude of the SCV were similar between protocols. The authors concluded that muscle fatigue is not a determining factor for the development of the SCV. Hopker and colleagues attested similar results. Participants completed either a non-metabolically stressful 100 intermittent drop-jumps protocol (pre-fatigue condition) or rest (control) for 33 minutes. The results of their study showed that locomotor muscle fatigue, tested by the reduction in power in the maximal voluntary cycling power test, was not associated with the development of the SCV^38^. Interestingly, the magnitude of the SCV was not significantly different between the two conditions despite significant differences in locomotor muscle fatigue. Recently, Dos Nascimento Salvador and colleagues published a study looking at the cause–effect relationship between the SCV and fatigue. They switched from constant work rate to isokinetic pedaling to quantify reductions in peak torque at three and eight minutes, with and without priming exercise. Results showed that the SCV after priming was reduced but there were no significant differences between conditions regarding the magnitude of the reduction of maximal isokinetic force and power at three and eight minutes^39^. This observation refutes a cause-effect relationship between fatigue and the development of the SCV.

However, the findings from this study are in contrast with the results by Keir and colleagues. Correlations were shown in both studies between some measures of fatigue and the SCV, however, a temporal association was only found in one study^9^. In one perspective, this difference highlights the importance of exercise intensity. Indeed, in the present study, step transition exercise was in the heavy domain, while the study by Keir and colleagues was in the severe domain^9^. In addition, the amplitude and type of fatigue was potentially different, as assessed by the difference in reduction of MVC after 18–20 minutes (9% for the present study vs 22% in the study by Keir and colleagues). If the SCV is related to fatigue parameters, it should be present in both exercise intensity domains. However, this was not the case, which suggests that the SCV may not be related to fatigue parameters.

The results in the present study are in agreement with results from a previous study regarding changes to velocity-specific peak power during cycling. Cannon *et al*.^17^ observed a reduction in velocity-specific peak power, which correlated with the SCV. However, as was observed in the present study, the reduction they observed was not temporally related to the development of the SCV. The reduction in velocity-specific peak power occurred prior to the SCV in their study, while excitation-contraction coupling was altered after the development of SCV in the present study. Nevertheless, both reported no changes during the development of the SCV which suggests that those alterations are likely not essential for the development of the SCV. If alterations to neuromuscular properties are not involved during the development of the SCV, at least during exercise in the heavy domain, it may be possible that the $\dot{{\rm{V}}}$O_2_ cost of force production may increase within a given fiber population. A progressive inhibition of ATP supply by anaerobic glycolysis, an increase in ATP usage per power output, and/or a reduction of ATP production per mole of oxygen (P/O_2_ ratio) are probably implicated in the SCV^40^. However, the documentation of a cause-effect relationship during exercise between muscle fatigue and reduced efficiency remains unknown.

### Experimental consideration

As with the study by Keir and colleagues (2016), at the end of exercise, the time to transfer the subject from the ergometer to the Biodex before the start of neuromuscular testing was less than one minute. One could argue that fatigue was already modified, and consequently the interpretation of the data in relation to fatigue during exercise is limited. Simply, fatigue is likely to have been underestimated in the present study and the measurement of fatigue during exercise would have been more appropriate. However, neuromuscular measurements were taken after a similar amount of time after each exercise, for each participant, and consequently, the change of the robustness of the relationship between fatigue and the SCV is likely to have been marginal, which should not change the general conclusions of the present study. Furthermore, the cause (fatigue) has to precede the effect (SCV); however, the data from the present study indicates that this was not the case. A further limitation is the fact that fatigue was measured during static contractions whereas cycling is a dynamic movement.

## Conclusion

Fatigue in the present study was observed during exercise completed at 30%∆ and which was at least 20 minutes in duration. Indirectly, these results suggest that the observed fatigue appears to be a result of impaired Ca^2+^ handling and/or reduced capability of cross-bridges to produce force. While significant correlations between the SCV relative to the primary phase and neuromuscular parameters were found, a temporal relationship between the development of the SCV and fatigue does not appear to exist. Therefore, it would seem that the alteration of neuromuscular properties in muscle is not required for the development of the SCV.

## Data Availability

The datasets generated and analysed during the current study are available from the corresponding author on reasonable request.
